# Supplementary material for: Acidic Microenvironment Enhances Cisplatin Resistance in Bladder Cancer via Bcl-2 and XIAP
Source: Curr Issues Mol Biol. 2025 Jan 10;47(1):43. doi: 10.3390/cimb47010043 (PMC11763506; doi:10.3390/cimb47010043)
Supplement: Supplementary file 1 [file cimb-47-00043-s001.zip › cimb-3415823-supplementary.pdf]

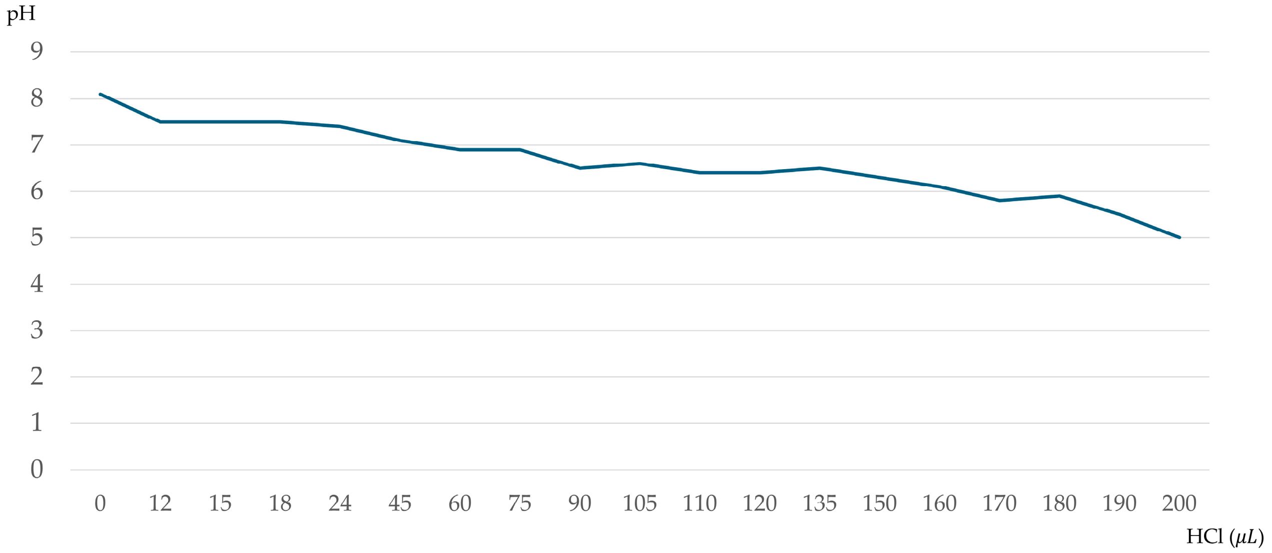

Figure S1: Correlation between pH of culture medium and hydrochloric acid;

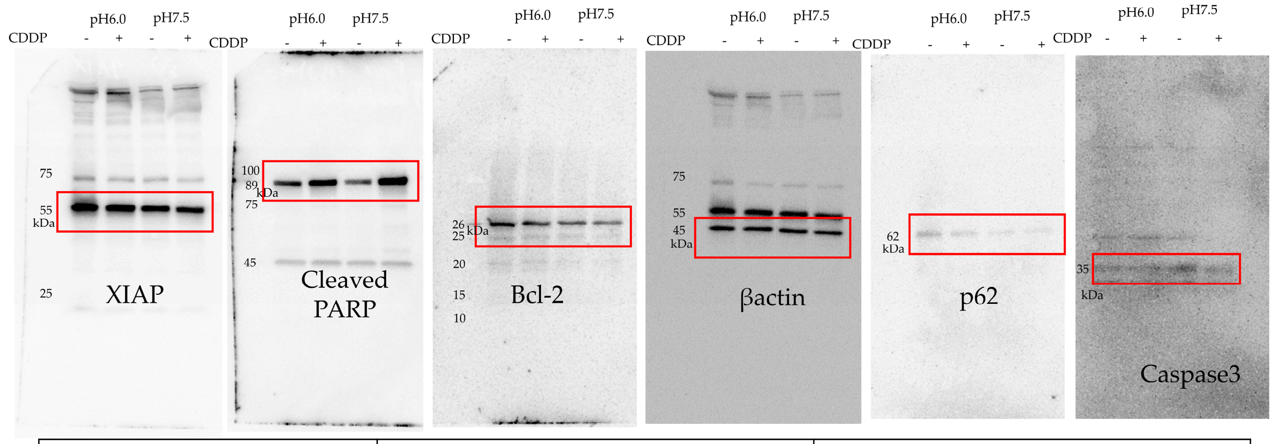

	pH6.0		pH7.5	
CDDP	-	+	-	+
XIAP/βactin	2.164999318	1.881275949	1.881275949	1.441597633
Cleaved PARP/βactin	0.933770379	1.34730156	0.633996872	1.861094598
Bcl-2/βactin	1.289502546	0.89302493	0.593680168	0.454970442
p62/βactin	0.982849011	0.581636989	0.278026942	0.238001205

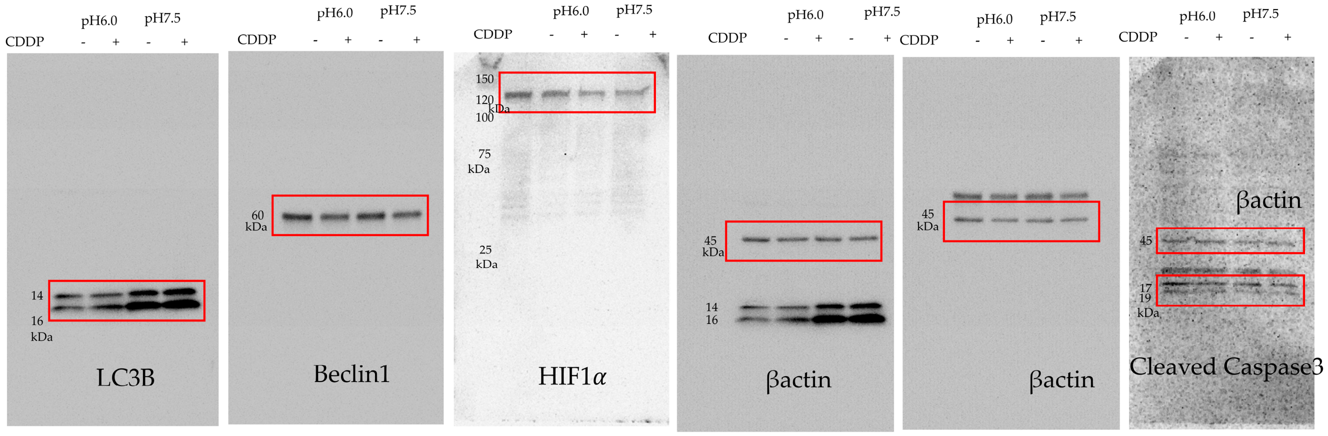

	pH6.0		pH7.5	
CDDP	-	+	-	+
LC3B/βactin	1.346389316	1.986953373	2.435289555	3.36536353
Beclin1/βactin	1.913657824	2.078203262	1.977492156	1.717769251
HIF1α/βactin	1.490510274	1.973083841	1.316508665	1.345720135
Caspase3/βactin	0.9216746	1.05888218	1.36299277	1.11373069
Cleaved Caspase3/βactin	1.05179183	1.03473488	1.09973338	1.20831753

Figure S2: Full-length images of Western blot analyses.
